# Liquid chromatography–mass spectrometry for measuring deoxythioguanosine in DNA from thiopurine-treated patients

**DOI:** 10.1016/j.jchromb.2016.06.017

**Published:** 2016-08-15

**Authors:** Sally A. Coulthard, Phil Berry, Sarah McGarrity, Azhar Ansari, Christopher P.F. Redfern

**Affiliations:** a Institute of Cellular Medicine, Newcastle University, Newcastle upon Tyne, UK; b Northern Institute of Cancer Research, Newcastle University, Newcastle upon Tyne, UK; c Gastroenterology Dept., East Surrey Hospital, Redhill, East Surrey, UK

**Keywords:** ALL, Acute childhood leukemia, 6-MP or MP, 6-mercaptopurine, AZA, azathioprine, IBD, inflammatory bowel disease, MeMP, methylmercaptopurine, MeMP-d3, Methymercaptopurine-D3, TIMP, thioinosine monophosphate, TIDP, thioinosine diphosphate, TITP, thioinosine triphosphate, TXMP, thioxanthine monophosphate, TGMP, thioguanosine monophosphate, TGDP, thioguanosine diphosphate, TGTP, thioguanosine triphosphate, IMPDH, inosine-monophosphate dehydrogenase, TPMT, thiopurine methyl-transferase, HGPRT, hypoxanthine-guanine phosphoribosyl transferase, ITPase, inosine tri-phosphatase, GMPS, guanosine monophosphate synthetase, dTG, deoxythioguanosine, TGN, thioguanine nucleotide, NBCs, nucleated blood cells, CD, Crohn’s disease, UC, ulcerative colitis, RBC, red blood cell, QC, Quality control, Azathioprine, Mercaptopurine thiopurine, Drug monitoring, Tandem mass spectrometry, LC–MS/MS, Thioguanine nucleotides, DNA

## Abstract

Adverse reactions and non-response are common in patients treated with thiopurine drugs. Current monitoring of drug metabolite levels for guiding treatment are limited to analysis of thioguanine nucleotides (TGNs) in erythrocytes after chemical derivatisation. Erythrocytes are not the target tissue and TGN levels show poor correlations with clinical response. We have developed a sensitive assay to quantify deoxythioguanosine (dTG) without derivatisation in the DNA of nucleated blood cells. Using liquid chromatography and detection by tandem mass spectrometry, an intra- and inter-assay variability below 7.8% and 17.0% respectively were achieved. The assay had a detection limit of 0.0003125 ng (1.1 femtomoles) dTG and was quantified in DNA samples relative to endogenous deoxyadenosine (dA) in a small group of 20 patients with inflammatory bowel disease, all of whom had been established on azathioprine (AZA) therapy for more than 25 weeks. These patients had dTG levels of 20–1360 mol dTG/10^6^ mol dA; three patients who had not started therapy had no detectable dTG. This method, comparable to previous methods in sensitivity, enables the direct detection of a cytotoxic thiopurine metabolite without derivatisation in an easily obtainable, stable sample and will facilitate a better understanding of the mechanisms of action of these inexpensive yet effective drugs.

## Highlights

• Thiopurines are effective immunosuppressant drugs.
• Monitoring of thiopurines is needed for research and clinical use.
• A sensitive assay of DNA-incorporated deoxythioguanosine is described.
• This method assays thiopurine nucleotides in DNA from nucleated blood cells.

## 1 Introduction

**Fig. 1:**
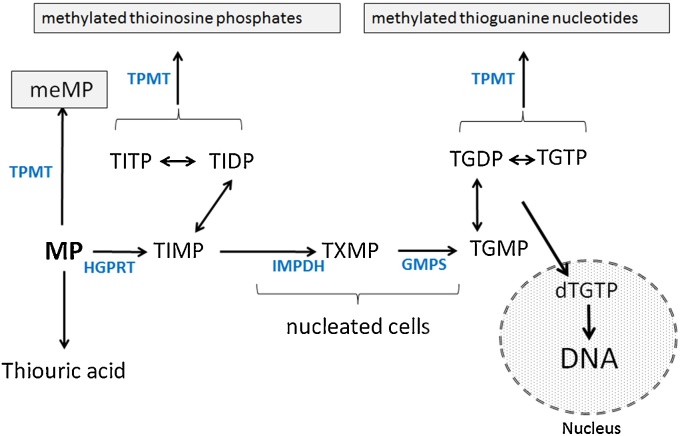
Schematic summarising the metabolism of 6- MP via the enzymes (Blue text) inosine-monophosphate dehydrogenase (IMPDH), thiopurine methyl-transferase (TPMT), hypoxanthine-guanine phosphoribosyl transferase (HGPRT), inosine tri-phosphatase (ITPase) and guanosine monophosphate synthetase (GMPS). MeMP, methylmercaptopurine; TIMP, thioinosine monophosphate with metabolism to the di- and triphosphates TIDP and TITP, respectively; TXMP, thioxanthine monophosphate; TGMP, thioguanosine monophosphate with metabolism to the di- and tri-phosphates TGDP and TGTP, respectively and incorporation into DNA (for interpretation of the references to colour in this figure legend, the reader is referred to the web version of this article).

Since their introduction into clinical practice more than six decades ago, the purine analogues mercaptopurine (MP; 3,7-dihydropurine-6-thione), azathioprine (AZA; 6-[3-methyl-5-nitroimidazol-4-yl]sulfanyl-7H-purine) and thioguanine (TG; 2-amino-3,7-dihydropurine-6-thione) have been used extensively in the treatment of diseases such as acute childhood leukaemia (ALL) [1], inflammatory bowel disease (IBD) [2], [3], auto-immune hepatitis [4] and rheumatoid arthritis [5]. Thiopurines require activation by hypoxanthine-guanine phosphoribosyl transferase (HGPRT, E.C. 2.4.2.8) followed by multi-step metabolism to TGNs or methylated products to exert their clinical effect (Fig. 1). Two key enzymes mediating thiopurine metabolism are inosine-monophosphate dehydrogenase (IMPDH) which is only present in nucleated cells [6], and thiopurine methyltransferase (TPMT) [7], [8], [9]. If treated with normal thiopurine doses, patients lacking TPMT activity develop high TGN levels that can lead to life-threatening leukopenia [10], [11], [12]. Cytotoxicity is mediated by a variety of mechanisms, including inhibition of *de novo* purine synthesis, disruption of G-protein signalling [13] and incorporation of thioguanine nucleotides (TGNs) into DNA with subsequent mismatching to thymidine, causing cell death by post-replicative mismatch repair [14], [15], [16].

Given a low therapeutic index and the wide variation in clinical response, including potential for life-threatening toxicity in patients with very low or absent TPMT activity, it is important to monitor and optimise thiopurine drug levels. Assays have been developed to measure metabolites in a range of cellular compartments including erythrocytes [17], [18], [19], [20], whole blood [21] and leukocyte DNA [22], [23] but ease of access to erythrocytes coupled with simple HPLC separation techniques has meant that quantifying thiopurine metabolites in erythrocytes has become the standard method for therapeutic monitoring. However, despite its value for assessing patient compliance, there is significant debate about concordance with therapeutic response in IBD [24], [25], [26]. Non-concordance can arise from methodological issues [27] and differences in metabolism between nucleated versus enucleated cells which lack the critical IMPDH enzyme. Therefore, the incorporation of deoxythioguanosine (dTG) into the DNA of nucleated cells may be a more relevant marker of therapeutic response. The aim of this study was to develop a sensitive assay for DNA-incorporated dTG in nucleated blood cells which could be developed for clinical use to study the mechanisms of response to thiopurines.

## 2 Materials and methods

### 2.1 Chemicals and enzymes

The dTG standard was from Carbosynth (Compton, UK); deoxyadenosine (dA) was from Sigma-Aldrich (Gillingham, UK) and deuterated 6-methylmercaptopurine (MeMP-d3) from Toronto Research Chemicals (Ontario, Canada). HPLC grade acetic acid was from Fisher Scientific (Loughborough, UK). Calf intestinal alkaline phosphatase and nuclease P1 from *Penicillium citrinum* were from Sigma-Aldrich, as were all other reagents.

### 2.2 Patient blood sample collection and processing

Clinical samples were from a small cohort of adult IBD patients (10 with Crohn’s Disease [CD] and 10 Ulcerative Colitis [UC]) treated with a range of doses of AZA, all of whom had been in clinical remission for more than 6 months with no therapeutic complications; three untreated IBD patients were used as controls. The study protocol was approved by NRES Committee South West − Cornwall & Plymouth, Bristol Research Ethics Committee Centre. DNA was isolated from whole blood collected in EDTA tubes, or from negative-control MOLT4 (T-acute lymphoblastic leukaemia) cells, using previously published methods [28]. Briefly whole blood was mixed with 3 vols of ice-cold buffer A (10 mM Tris, 320 mM sucrose, 5 mM Mg Cl_2_ 1% Triton × 100 pH 8) and centrifuged at 1730*g* for 10 min at 4 °C. The supernatant was removed and the remaining pellet re-suspended in 1 mL buffer B (400 mM Tris, 60 mM EDTA, 150 mM NaCl and 1% SDS pH 8) plus 0.5 mL of 5 M sodium perchlorate, mixed for 10 min, then incubated at 65 °C for 45 min. To this, 2.5 mL of chloroform was added and mixed for 20 min prior to centrifugation at 432*g* for 10 min at 4 °C. The top layer was removed and 2.5 vols of ethanol added to precipitate the DNA which was spooled out, air-dried and re-suspended in 100–200 μL of double-deionised water. The red blood cell (RBC) TGN assays were performed by a commercial laboratory at the City Hospital Birmingham (cityassays.org). Other tests were part of routine clinical care at the six hospitals contributing samples.

DNA was digested with P1 nuclease and alkaline phosphatase to release nucleosides for LC–MS/MS analysis using previously-described methods [29]. Briefly, samples were prepared in the following manner: 5 μg DNA in a total volume of 100 μL double-deionised water, containing 124.38 ng/mL (0.735 μM) Methymercaptopurine-D3 (MeMP-d3) as an internal standard to control for extraction efficiency, was denatured by heating to 100 °C for 5 min. After chilling on ice for 2 min, 10 μL of 10X digestion buffer (500 mM sodium acetate, 10 mM MgCl_2_ pH 5.3) and 5 μL of 0.12U/μL nuclease P1 was added and incubated for 1 h at 50 °C. Finally, 20 μL of 1 M Tris-HCl and 1 μL of alkaline phosphatase (1U/mL) were added to each sample and incubated for 30 min at 40 °C. MeMP-d3 was used as an internal standard as it was the only deuterated thiopurine metabolite available commercially at the time.

### 2.3 LC–MS/MS analysis of thioguanine incorporated into DNA

Chromatographic separation of dTG, dA and MeMP-d3 was achieved using a Prominence HPLC (Shimadzu, Kyoto, Japan) with an XSelect HSS T3 4.6 × 100 mm 3.5 μm and a VanGuard cartridge 3.9 × 5 mm 3.5 μm guard column (Waters, Massachusetts, USA) maintained at 30 °C. Analytes were eluted with HPLC grade (Sigma-Aldrich) mobile phases comprising 0.05% aqueous formic acid (A) and 0.05% formic acid in acetonitrile (B). The flow rate was 0.5 mL/min and the mobile phase system consisted of a starting condition of 1% buffer B increasing to 3% at 1.1 min, 8% at 2.4 min and increasing to a maximum of 30% at 4.1 min then decreasing to 5% at 4.5 min, maintained until 5.5 min then decreasing to 1% for an equilibration period of 2.5 min. An API4000 triple quadrupole LC–MS/MS (Applied Biosystems, California, USA) was used for analysis with electrospray ionisation performed in positive ion mode using nitrogen gas with the following optimum settings: curtain gas, 20; ion source gas 1, 10; ion source gas 2, 10; ion spray voltage, 5500; collision gas, 6; entrance potential, 10; ionisation temperature, 300∘C. Mass transitions and optimised MS/MS parameters for analyte quantification are summarised in Table 1.

Standards were prepared at 100,000 ng/mL dA and 2000 ng/mL dTG before serial dilution in double-deionised water to 390 ng/mL dA and 3.9 ng/mL dTG, respectively, after which dA standards were diluted 1/100 and dTG standards 1/10 in double-deionised water prior to injection. Quality controls (QCs) were diluted using 10 × digestion buffer as for patient samples at three concentrations; these were then diluted either 1/10 (for dTG) or 1/100 (for dA) in double-deionised water to yield concentrations of high (40,000 ng/mL dA: 400 ng/mL dTG), medium (4000 ng/mL dA: 40 ng/mL dTG) and low (400 ng/mL dA: 4 ng/mL dTG) concentration standards prior to injection.

**Fig. 2:**
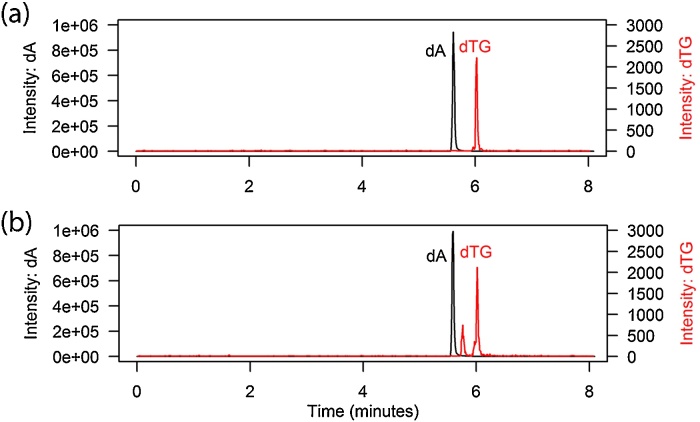
LC–MS/MS profiles (50 μL injection volume) for (a) dA and dTG standards (125 ng/mL dA and 0.78 ng/mL dTG; peak areas represent 6.25 ng dA and 0.039 ng injected dTG), and (b) a patient on treatment with azathioprine. The additional small peak in (b) between the dA and dTG peaks is thioguanosine. The retention time of the internal standard MeMP-d3 (not shown) was 6.92 min.

Standards and controls, as for patient samples, contained 124.38 ng/mL MeMP-d3 prior to 1/10 or 1/100 dilution. Samples were diluted in double-deionised water 1/10 (for dTG determination) and 1/100 (for dA) to avoid signal saturation on the mass spectrometer. Sample injections represented 0.2 μg patient DNA per injection for dTG determination and 0.02 μg DNA for dA. Standards were analysed in duplicate, QCs in triplicate, and negative control MOLT4 DNA and samples were analysed in singlicate, all with a 50 μL injection volume. Samples where the internal standard was outside an acceptable range (within 15% of the expected value) were discarded. For measurement of dTG, background at the retention time for dTG in extracts of negative control MOLT4 DNA (loaded as for patient DNA) was subtracted from the dTG peak in patient samples (Fig. 2).

### 2.4 LC–MS/MS validation

Method validation was adapted from the 2001 FDA guidelines [30]. Not all validation parameters could be determined because calibrators consisting of dTG incorporated into DNA are not obtainable and it is not possible to have a processed sample containing the DNA matrix without the presence of dA.

#### 2.4.1 Selectivity

As dA is always present in processed DNA samples, the absence of dTG in negative-control MOLT4 cells and in patients not on thiopurine therapy indicates selectivity.

#### 2.4.2 Accuracy and precision

QC solutions of analytes were prepared in DNA digestion buffer at the concentrations 400, 40 and 4 ng/mL for dTG and 40,000, 4000 and 400 ng/mL for dA, equating to 2, 0.2 and 0.02 ng for dTG and 20, 2 and 0.2 ng for dA per injection, and used to determine intra- (5 separate analytical runs containing 5 QCs per concentration) and inter-assay (10 QCs per concentration) accuracy and precision from multiple injections (Table 2).

#### 2.4.3 Recovery and sample dilution

It is not possible to obtain matrix free of dA, and the addition of exogenous calibrators would not be a suitable equivalent measure of the efficiency of extraction from DNA; therefore, we could not perform a full recovery analysis. The testing of different DNA extracts from the same sample and different amounts of DNA from the same sample gave a stable ratio between dA and dTG (Table 3) and this indicates that extraction efficiency is maintained across both analytes.

#### 2.4.4 Freeze and thaw stability

QC solutions of analytes were prepared in DNA digestion buffer as described above at the concentrations 400, 40 and 4 ng/mL for dTG and 40,000, 4000 and 400 ng/mL for dA, stored at −20 °C and subjected to between 1 and 3 freeze thaw cycles, each of 24 h duration. The QCs were then thawed and analysed on the same analytical run following the third freeze thaw cycle. All results were within 15% of expected values (Table 4).

#### 2.4.5 Short-Term temperature stability

QC solutions of analytes were prepared in DNA digestion buffer at the concentrations 400, 40 and 4 ng/mL for dTG and 40,000, 4000 and 400 ng/mL for dA and tested after 24 h incubation at 4 °C (Table 5).

### 2.5 Statistical analysis

Data were analysed using R [31]. Residuals from linear and non-linear models of dTG versus drug dose were not normally distributed (Shapiro-Wilks test, P < 0.01) and, therefore, non-parametric Kendall’s rank correlation [32] was used for all analyses.

## 3 Results

We report here a sensitive LC–MS/MS method for measuring, without derivatisation, the thiopurine metabolite, dTG, in DNA isolated from whole blood of patients with CD or UC treated with AZA (Fig. 2). The method range was from 0.0975 ng to 50 ng injected for dA and from 0.000625 ng to 10 ng injected for dTG, with R^2^ > 0.99 for the standard curves (Table 2). There was no matrix effect and no concentration effect on the ratio between dTG and dA (Table 3). Three quality controls containing standards made in digestion buffer at low, medium and high concentrations within the standard curve range (duplicates at each concentration per run) were included in all runs; the intra-day variation was <7.8% and 11.9% for dA and dTG, respectively, and the inter-day variation was <17.0% and 15.9% for dA and dTG, respectively (Table 2). The limit of detection (LOD) for dTG standards was 0.0003125 ng and lowest limit of quantification (LLOQ) 0.000625 ng (Table 2). In the patient samples, dA was at very high concentrations and, therefore, in the standard mixtures dA was used at 100-fold higher levels than dTG to achieve appropriate standard curve ranges for both analytes, and samples were diluted 10-fold for dA determination relative to dTG determination. Incorporation of dTG into the DNA was reported as moles dTG per 10^6^ moles dA, after subtraction of background at the dTG retention time using digested DNA from untreated MOLT4 cells, and was detectable in 0.2 μg patient DNA per LC–MS/MS injection. Patients included were established on AZA therapy for more than 6 months and were in clinical remission according to the treating physician. For this cohort of patients, the incorporated dTG levels varied from 20 to 1360 mol dTG/10^6^ moles dA; expressed in the units used by Jacobson et al. [33], this represents 14–940 fmol/μg DNA (median 113; Table 6). Within this small group of patients there was no significant positive correlation between dTG levels and drug dose (Kendall’s rank correlation coefficient P = 0.143), although further studies with a larger sample size are warranted. There were no indications for potential relationships between dTG levels and RBC TGN measurements (Kendall, P = 0.85) or TPMT activity (Kendall, P = 0.72). Patients who were not on AZA treatment had no detectable dTG in their DNA (Table 6).

## 4 Discussion

The method presented here has good sensitivity for dTG detection in DNA from whole blood with dTG levels expressed relative to an endogenous reference (dA) for sample loading; only 0.2 μg DNA was required per injection, with intra- and inter-assay calibration provided by commercially available standards. Using this approach, we were able to quantify DNA-incorporated dTG directly in the DNA of adult patients with IBD. Jacobsen et al. [33] have developed a similar assay but this requires derivatisation before analysis, necessitating standard dTG-incorporated DNA to control for variation in the derivatisation reaction. Nevertheless, both methods appear to have comparable sensitivity with respect to analyte detection.

For the samples from children with ALL treated with MP Jacobson et al. [33] reported incorporated dTG amounts of 45–1190 fmol TG/μg DNA (median 377). Ebbesen et al. [34] reported similar values for standard- and intermediate risk childhood ALL patients on the NOPHO-ALL2008 protocol [35]. For the adult IBD patients reported here, DNA-incorporated dTG quantities, expressed in the same units, were 14–940 fmol/μg DNA. The MP dose (NOPHO-ALL2008 protocol [35]) received by patients whose samples were used by Ebbesen et al. [34] was similar to guidelines for adult IBD patients [36]. Therefore, incorporated dTG amounts were, on the whole, comparable in these childhood ALL and adult IBD patients using different methods, despite differences in disease biology and patient age. Using their derivatisation assay [33], Ebbesen et al. [34] reported that DNA-TGNS were independent of TPMT status in childhood/adolescent ALL patients on maintenance therapy with 6-MP. This was also the case for the IBD patients investigated here, but, unlike Jacobson et al. [33], we found no correlation between RBC TGN levels and DNA-incorporated dTG in whole blood DNA of IBD patients. Such a lack of correlation is not surprising given the very different cellular compartments and the fact that clinical studies have shown major difference between RBC TGNs and leukocyte cytosolic TGNs in the same blood samples [37], [38]. Disease biology may also be an important factor and it is critical to understand thiopurine metabolism in relevant target tissues and how this relates to pharmacological markers and clinical responses. With the method described here, sample processing, DNA isolation, digestion and analysis are simple and easily set up in diagnostic laboratories, and this will facilitate a greater understanding of thiopurine pharmacology which is essential if these inexpensive yet effective drugs are to be used to their full potential.

## Figures and Tables

**Table 1:** Mass transitions and optimised MS/MS parameters for analyte quantification, deoxythioguanosine (dTG), deoxyadenosine (dA), Methymercaptopurine-D3 (MeMP-d3).

Analyte	Analyte Retention Time (min)	MRM transition (*m*/*z*)	Declustering Potential (V)	Entrance Potential (V)	Collision Energy (eV)	Collision exit potential (V)
dTG	6	284.19 −> 168.2	41	10	15	10
dA	5.6	252.30 −> 136.2	31	10	21	8
MeMP-d3	6.9	170.1 −> 152.2	66	10	33	10

**Table 2:** Standard curve and quality control analyses.

Analyte	dA	dTG
LOD (ng injected) a	nd^f^	0.0003125
LLOQ (ng) b	nd^f^	0.000625
*analytica*l range (ng injected)	0.0975 ng to 50 ng	0.000625 ng–10 ng
average curve^c^	Y = −6218126 ×^2^ + 26200326x + 5014429	y = 263992 × + 148.9
weighting	1/x^2^	1/x
fit	quadratic	linear
Intra-day High QC^d^	3.5%	4.2%
Intra-day Mid QC	7.8%	7.7%
Intra-day Low QC	3.3%	11.9%
Inter-day High QC^e^	7.8%	8.2%
Inter-day Med QC	5.1%	8.5%
Inter-day Low QC	17.0%	15.9%

a Limit of detection (10 × baseline).

b Lower Limit of Quantification (2 × LOD).

c Calibration curves adjusted R^2^: dA = 0.991; dTG = 0.996.

d Intra-day, n = 10.

e Inter-day, n = 5.

f nd, not determined: dA standard concentrations were 100-fold higher than for dTG and sensitivity for dA was not relevant.

**Table 3:** Consistent results using different starting amounts of DNA for digestion from the same sample. N/D, not detected.

Amount of DNA digested (μg)	ng dA^a^ detected	ng dTG^b^	Moles dTG/10^6^ moles dA
5	10.1	0.059	520.7
2.5	5.6	0.031	493.4
1.25	2.5	0.013	461.9
0.625	1.4	0.006	398.2
Untreated control patient	8.5	N/D	N/D

DNA range	mean^c^	Stdev	CV
1.25 − 5ug	49.20	2.94	6%
0.625 − 5ug	46.86	5.27	11%

a Representing 0.018 μg DNA injected.

b Representing 0.18 μg DNA injected.

c Moles dTG/10^6^ moles dA.

**Table 4:** Freeze-thaw (FT) stability.

	dA			dTG		
	LQC	MQC	HQC	LQC	MQC	HQC
FT1^a^	0%	0%	0%	0%	0%	0%
FT2^a^	−3%	12%	4%	1%	−1%	8%
FT3^a^	−6%	4%	9%	−10%	−13%	−12%
%CV across all samples	3%	6%	5%	6%	8%	11%

^a^Signal change compared to FT cycle 1.

**Table 5:** Low temperate stability.

	Moles dTG/10^6^ moles dA		% difference between days
	Day 1	Day 2 (Overnight @4 °C)	
LQC	105.231	88.495	−16%
MQC	66.707	68.164	2%
HQC	77.813	86.026	10%

**Table 6:** Quantification of incorporated dTG (amount detected in 50 μL sample, representing 0.18 μg DNA, injected onto column) in NBC in relation to clinical parameters from patients (ID 1–20) treated with azathioprine (dose) and three untreated patients (A–C). ID, patient identifier; N/A, data not available; N/D, analyte not detected.

ID	Disease^a^	dose	ng dA^b^	ng dTG	moles dTG/10^6^ moles dA	fmoles dTG/μg DNA	WCC^c^	Neut^c^	ALT^c^	AP^c^	TPMT	RBC TGN^c^	RBC MMP^c^
1	CD	100	3.97	0.003	59.2	52.0	10.9	9.4	10	60	70	N/A	N/A
2	UC	150	9.25	0.035	333.2	682.2	5.4	3.3	20	50	70	N/A	N/A
3	UC	150	3.115	0.001	20.2	13.9	4.3	3	29	89	87	N/A	N/A
4	UC	125	7.4	0.011	135.4	221.8	3.8	2.4	5	42	14	N/A	N/A
5	CD	200	4.62	0.027	524.1	535.9	3.4	2.2	21	37	70	N/A	N/A
6	UC	150	4.555	0.005	97.4	98.2	9.3	5.1	16	94	123	N/A	N/A
7	CD	150	14.7	0.022	133.3	433.8	5.5	3.6	13	52	102	N/A	N/A
8	UC	100	6.1	0.003	36.3	49.1	5	3	13	70	78	N/A	N/A
9	CD	100	4.185	0.004	74.2	68.7	9	8	11	63	80	N/A	N/A
10	CD	50	7.35	0.002	26.5	43.2	10.5	8.3	18	91	102	N/A	N/A
11	UC	150	11.4	0.006	46.7	117.8	8	4	15	86	81	148	412
12	UC	200	6.2	0.013	178.8	245.4	5.8	4.1	22	71	N/A	296	6698
13	CD	42	11.95	0.024	181.1	479.0	5	2.9	14	16	N/A	522	0
14	UC	25	5.15	0.008	129.2	147.2	3.8	2.7	16	78	N/A	318	0
15	CD	200	6.45	0.002	27.5	39.3	9.2	9	8	80	94	559	0
16	CD	150	4.77	0.005	89.3	94.2	8	5.7	16	64	92	120	221
17	UC	175	1.785	0.019	921.7	364.2	7.8	5.7	13	58	N/A	293	363
18	UC	100	7.2	0.032	393.6	627.2	3.3	2.4	15	80	116	477	219
19	CD	75	14.45	0.007	44.5	142.3	6.3	4	15	100	100	80	320
20	CD	200	3.125	0.048	1359.5	940.3	5.1	3	28	45	94	169	6398
A	UC	0	10	N/D	N/D	N/D	4.6	3.2	26	202	126	N/A	N/A
B	UC	0	9.6	N/D	N/D	N/D	3.6	2.3	19	52	134	N/A	N/A
C	CD	0	8.45	N/D	N/D	N/D	12.3	10.2	20	85	81	N/A	N/A

a Crohn’s Disease, CD; Ulcerative colitis UC.

b Representing 0.018 μg DNA injected.

c Normal ranges: White cell count (WCC), 4–11 × 10^9^/L; Neutrophils (Neut), 2.5–7.5 × 10^9^/L; Alanine aminotransferase (ALT), 7–56 U/L; Alkaline Phosphatase (AP), 44–147 IU/L; TPMT, 70–110 mU/L; RBC TGNs, reference range 235–450 pmol/8 × 10^8^ RBC.
